# Decreased Vessel Density in Retinal Capillary Plexus and Thinner Ganglion Cell Complex Associated With Cognitive Impairment

**DOI:** 10.3389/fnagi.2022.872466

**Published:** 2022-04-26

**Authors:** Kai Yang, Lele Cui, Xueyu Chen, Chuang Yang, Jingwei Zheng, Xiaoxuan Zhu, Yunfan Xiao, Binbin Su, Chunmei Li, Keai Shi, Fan Lu, Jia Qu, Ming Li

**Affiliations:** ^1^Eye Hospital and School of Ophthalmology and Optometry, National Clinical Research Center for Ocular Diseases, Wenzhou Medical University, Wenzhou, China; ^2^Department of Biostatistics, School of Public Health, Cheeloo College of Medicine, Shandong University, Jinan, China; ^3^Department of Mental Health, The First Affiliated Hospital of Wenzhou Medical University, Wenzhou, China

**Keywords:** cognitive impairment, optical coherence tomography angiography, retinal capillary plexus, ganglion cell complex, imaging

## Abstract

**Background:**

To determine the association of the retinal capillary plexus (RCP) and ganglion cell complex (GCC) with cognitive impairment using optical coherence tomography angiography (OCTA).

**Methods:**

A cross-sectional, community-based study utilizing data from the participants enrolled between August 2019 and January 2020 in the Jidong Eye Cohort Study. We assessed the vessel density in RCP and GCC thickness using OCTA, and cognitive testing using the Montreal Cognitive Assessment (MoCA). Cognitive impairment in this study was defined as MoCA score < 24. We used multivariable analysis to evaluate the association of RCP and GCC with cognitive impairment after adjusting for confounders.

**Results:**

This study analyzed 1555 participants. The mean age of participants was 52.3 (8.4) years, and 861 (55.4%) were women. Cognitive impairment was observed in 268 (17.2%) participants. The adjusted odds ratio (OR) with 95% confidence interval (95% CI) for parafovea vessel density in the deep RCP with cognitive impairment was 1.20 (1.03–1.39). For vessel area and length density surrounding foveal avascular zone with cognitive impairment, the ORs with 95% CIs were 1.23 (1.07–1.41) and 1.30 (1.13–1.49), respectively. For thickness in the superior GCC with cognitive impairment, the OR with 95% CI was 1.16 (1.01–1.32).

**Conclusion:**

Lower vessel density in the RCP and thinner GCC were associated with cognitive impairment. Our results suggest that alterations in the RCP and GCC could provide further evidence when assessing the cognitive function and may even be potentially useful biomarkers in the detection of cognitive impairment.

## Introduction

Dementia has become a worldwide public health issue because of rapidly aging populations (Reitz et al., 2011). In 2015, dementia reportedly affected approximately 46 million people worldwide, and this number is estimated to reach 130 million people by 2050 (Baumgart et al., 2015). Mild cognitive impairment is deemed as the prodromal stage of dementia and persons with mild cognitive impairment are more likely to convert to dementia (Ding et al., 2015; Jack et al., 2018). Thus, identifying individuals in the early stage of cognitive impairment is necessary for the prevention of dementia (Koepsell and Monsell, 2012; Livingston et al., 2020).

Currently, there remain unmet needs for biological markers for identifying individuals in the early stage of cognitive impairment (Snyder et al., 2020). Amyloid positron emission tomography neuroimaging and cerebrospinal fluid testing are limited by their availability, invasiveness, time requirement, and high costs (Snyder et al., 2020). Moreover, although substantial progress has been made for blood-based biomarkers in the past decade, issues related to standardization and replication persist (O’Bryant et al., 2017; Hampel et al., 2018; Shi et al., 2018). There is homology between cerebral system and retina due to the shared diencephalic origin, and the retina has been deemed as an extension of the central nervous system (London et al., 2013). One potential screening test is retinal imaging using optical coherence tomography (OCT) (Gupta et al., 2020; Snyder et al., 2020). OCT is a non-invasive, rapid imaging tool that can produce cross-sectional retinal images and accurately measure the thickness of retinal components (Huang et al., 1991; Jiang et al., 2018a; Ito et al., 2020). Recently, OCT has evolved from structural OCT to OCT angiography (OCTA), which enables better visualization and quantitative analysis of the retinal capillaries (Spaide et al., 2018; Wang et al., 2021). Furthermore, OCTA images can be obtained in parallel with OCT images using the same instrument.

Numerous studies using OCT have shown that a relatively thinner ganglion cell complex (GCC) or retinal nerve fiber layer (RNFL) was associated with cognitive impairment (Cheung et al., 2015; Jones-Odeh and Hammond, 2015; Ko et al., 2018; Ito et al., 2020). However, only case-control and hospital-based studies using OCTA have investigated the vascular alternations in patients with AD using OCTA (Zhang et al., 2020; Cheung et al., 2021; Peng et al., 2021). Findings from those studies were inconsistent and the effects of important sub-categorizations (i.e., sex, diabetes, hypertension) remain unknown (Rifai et al., 2021). Studies have shown that vascular health factors, such as sex, diabetes, and hypertension, may also affect the retina–brain association, as they are crucial determinants of both retinal vascular changes and dementia risk (You et al., 2019; Livingston et al., 2020).

Therefore, this study aimed to investigate the association of the retinal capillary plexus (RCP) and GCC with cognitive impairment in a community-based setting using OCTA.

## Materials and Methods

### Study Design and Participants

**FIGURE 1:**
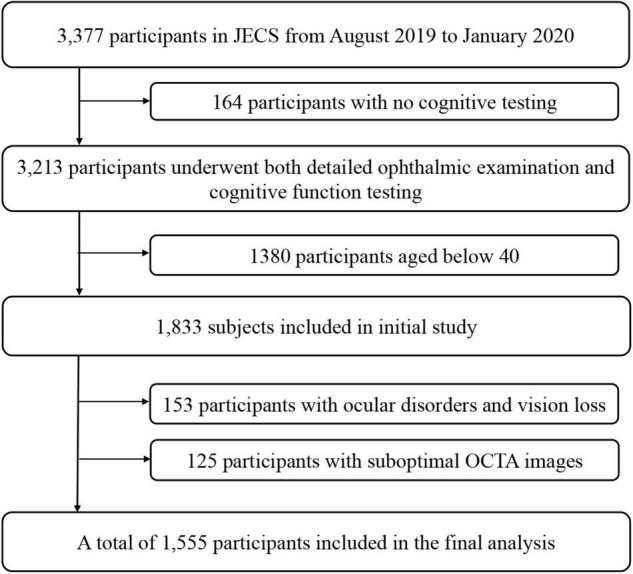
Flow Chart of the Study Population. JECS, Jidong Eye Cohort Study; OCTA, optical coherence tomography angiography.

In this study, we analyzed data of the Jidong Eye Cohort Study. Details of the design and methods have been previously described (Yang et al., 2020). Participants in this study were recruited from the Jidong communities (Tangshan city, northern China) from August 2019 to January 2020. In this study, we only entry the participants aged over 40 in this study, because the cognitive impairment occurrence in participants aged below 40 is rare (16/1380). A total of 1833 participants aged over 40 underwent both detailed ophthalmic examination and cognitive function testing (Song et al., 2020). The following participants were excluded from the study: 153 for ocular disorders and vision loss; 125 for suboptimal OCTA images. And no participants in our study had a history of dementia. The analyses ultimately included data of 1555 participants (Figure 1). Among these, 268 (17.2%) displayed poor performance on the Montreal Cognitive Assessment (MoCA) and were categorized into the cognitive impairment group. The remaining 1287 (82.8%) participants were included in the normal group.

This study followed the Declaration of Helsinki guidelines and was approved by the Ethics Committee of Staff Hospital of Jidong oil-field of Chinese National Petroleum (approval document 2018 YILUNZI 1). All participants provided written informed consent.

### General Variable Assessment

In this study, we obtained the basic information on the participants using laboratory tests, clinical examinations, and standardized questionnaires for age, sex, income, education level, current smoker status, and current drinker status (Zhang et al., 2013; Xia et al., 2020). Face-to-face interviews were performed by well-trained examiners. We divided the average monthly income into “<¥3000,” “¥3000–5000,” and “>¥5000.” We categorized the education levels as “illiteracy or primary,” “middle school,” and “college graduate or above.” In our study, hypertension was defined as systolic blood pressure ≥ 140 mmHg, or diastolic blood pressure ≥ 90 mmHg, or current use of antihypertension medication, or self-reported history of hypertension. Diabetes was defined as fasting blood glucose (FBG) ≥ 7.0 mmol/L, or current use of antidiabetic medication, or self-reported history of diabetes. Dyslipidemia was defined as low-density lipoprotein (LDL-C) ≥ 3.37mmol/L, or high-density lipoprotein (HDL-C) < 1.04mmol/L, or total cholesterol (TC) ≥ 5.18mmol/L, or triglyceride (TG) ≥ 1.7mmol/L, or current use of lipid-lowering therapy, or self-reported history of dyslipidemia (Chen et al., 2019).

### Ophthalmic Examination

We measured best-corrected visual acuity using standard logarithmic visual acuity charts at a distance of 5 m from participants. Vision loss in this study was defined as best-corrected visual acuity < 0.5 logMAR. We obtained the refractive status using an auto refractometer (KR800; Topcon; Tokyo, Japan). We calculated the refraction as the spherical equivalent (spherical value and half of the cylindrical value). We acquired the digital fundus photographs of both eyes using a 45° non-mydriatic fundus camera (CR2AF; Canon; Tokyo, Japan). At least two independent ophthalmologists identified ocular disorders (i.e., nerve fiber layer defects, macular degeneration, retinal vascular occlusion, and diabetic retinopathy) and vision loss. We excluded participants with ocular disorders and vision loss from the analyses.

### Optical Coherence Tomography Angiography Assessment

We acquired OCTA images using a spectral-domain OCTA (RTVue XR Avanti with AngioVue; Optovue; Fremont, CA, United States). OCTA technology has been previously described in greater detail (Spaide et al., 2018). OCTA image acquisition was performed using a 3 × 3 mm^2^ scan in the macula at a scan density of 304 × 304 A-scans with no pupillary dilation. We used the Early Treatment Diabetic Retinopathy Study grid as follows: two concentric circles with diameters of 1 and 3 mm divided into six quadrants (fovea, parafovea, superior, inferior, nasal, and temporal). The regionals of parafovea served as representatives of average RCP vessel density and average GCC thicknesses in this report due to the special structure of foveal area, although we also presented other regionals. The commercially available AngioAnalytics software (version 2017.1.0.155) automatically provided the vessel density (the proportion of the flow signals within the range of predefined area and depth) of superficial RCP (vessels between the internal limiting membrane and inner plexiform layer) and deep RCP (vessels between the inner and outer plexiform layer) in the macula. We also acquired the foveal avascular zone area (FAZ) and perimeter (PERIM), acircularity index (AI), vessel area density (FD300 AD), and length density (FD300 LD) within a 300 μm width ring surrounding the FAZ from the software automatically. The internally installed software automatically provided the GCC thickness (layer from the internal limiting membrane to the inner plexiform layer), as well.

The OCTA device included a three-dimensional projection artifact removal algorithm to reduce motion artifacts and increase image quality (Kraus et al., 2012). We only included the OCTA images with a signal strength index ≥ 8 and excluded the images with defects (i.e., significant motion artifacts, local weak signal, and incorrect segmentation) from the study. If available, we used the measurement of the right eye for each individual, whereas we used the left eye in the absence of valid data for the right eye (Ito et al., 2020; Ward et al., 2020).

### Assessment of Cognitive Function

To test for cognitive function, all participants underwent standardized neurological assessments by experienced neuropsychologists using the Montreal Cognitive Assessment (MoCA) and Mini-Mental State Examination. In this study, we use the MoCA to evaluate cognitive impairment as the MoCA is more sensitive and the specificity is not significantly different from others (Kasten et al., 2010; Dong et al., 2012). MoCA is a scale that evaluates visuospatial and executive functions, naming, language, attention, confrontation, memory, and abstraction (Nasreddine et al., 2005). Participants can obtain a maximum of 30 points; lower scores indicate lower cognitive function. If the participant had ≤12 years of education, we increased the total test score by one point. In this study, a score of ≥24 points was used to indicate normal cognitive function (Potocnik et al., 2020). We expressed cognitive function as a continuous MoCA score or a dichotomization of MoCA score as being cognitive impairment or normal.

### Statistical Analysis

We calculated the means and standard deviations for the continuous variables. We tested the differences in continuous variables between the groups using the *t*-test for normally distributed variables. We described the categorical variables using frequencies and percentages and examined them using the chi-square or Fisher’s exact test. Missing data were deemed as missing at random.

We performed multivariable models to estimate the association between vessel densities in the RCP and GCC thicknesses and cognitive impairment. Multivariable logistic regression model was used for binary cognitive impairment or normal MoCA score and multivariable linear regression model for continuous MoCA score. We adjusted all multivariable models for age, sex, educational status, income, refraction, hypertension, diabetes, and dyslipidemia. We also tested the interaction effects for the association of the RCP and GCC with cognitive impairment within the subgroups (sex, diabetes, hypertension, age) by including interaction terms in the adjusted model using all subjects in our study.

We measured the association as odds ratios (ORs) or Betas and 95% confidence intervals (CIs) per standard deviation decrease. We considered statistical significance as a *P*-value < 0.05. We performed all statistical analyses using the SAS software, version 9.4 (SAS Institute Inc., Cary, NC, United States).

## Results

### Baseline Characteristics of Participants

A total of 1555 participants were included in the analyses. The mean age of the included participants was 52.3 (8.4) years, and 861 (55.4%) were women. Cognitive impairment was observed in 268 (17.2%) participants. Table 1 shows the baseline characteristics of the cognitive impairment (MoCA < 24) and normal (MoCA ≥ 24) groups.

**TABLE 1:** Baseline characteristics of eligible participants in the study.

Characteristics	Total (*n* = 1555)	Cognitive impairment (*n* = 268)	Normal (*n* = 1287)	*P*-value
Age, y, mean (SD)	52.3 (8.4)	58.3 (8.3)	51.0 (7.8)	<0.001
Sex, n (%)				0.55
Male	694 (44.6)	124 (46.3)	570 (44.3)	
Female	861 (55.4)	144 (53.7)	717 (55.7)	
Educational level, *n* (%)				<0.001
Illiteracy/Primary School	66 (4.2)	37 (13.8)	29 (2.3)	
Middle School	698 (44.9)	151 (56.3)	547 (42.5)	
College/University	791 (50.9)	80 (29.9)	711 (55.2)	
Income, n (%)				<0.001
<¥3,000	192 (12.4)	70 (26.1)	122 (9.5)	
¥3,000–5,000	1336 (85.9)	197 (73.5)	1139 (88.5)	
> ¥5,000	27 (1.7)	1 (0.4)	26 (2.0)	
Current Smoking, *n* (%)	300 (19.4)	58 (21.6)	242 (18.9)	0.30
Current Drinking, *n* (%)	232 (15.1)	46 (17.3)	186 (14.7)	0.28
Hypertension, *n* (%)	575 (37.0)	125 (46.6)	450 (35.0)	<0.001
Diabetes, *n* (%)	193 (12.4)	47 (17.5)	146 (11.3)	0.005
Dyslipidemia, *n* (%)	1119 (72.0)	216 (80.6)	903 (70.2)	<0.001
Systolic pressure, mmHg, mean (SD)	128.1 (17.5)	133.5 (18.4)	127.0 (17.0)	<0.001
Diastolic pressure, mmHg, mean (SD)	81.7 (12.7)	82.2 (12.6)	81.6 (12.8)	0.51
FBG, mmol/L, mean (SD)	5.7 (1.6)	6.0 (1.7)	5.7 (1.5)	0.003
BMI, kg/m^2^, mean (SD)	24.7 (3.2)	25.0 (3.1)	24.6 (3.2)	0.11
TC, mmol/L, mean (SD)	5.2 (1.0)	5.3 (1.0)	5.2 (1.0)	0.10
Triglycerides, mmol/L, mean (SD)	1.8 (1.3)	1.8 (1.2)	1.8 (1.3)	0.95
HDL-C, mmol/L, mean (SD)	1.2 (0.3)	1.2 (0.3)	1.2 (0.3)	0.32
LDL-C, mmol/L, mean (SD)	2.2 (0.8)	2.2 (0.8)	2.2 (0.8)	0.46
Refraction, diopter, mean (SD)	−1.0(2.3)	−0.1(1.8)	−1.2(2.4)	<0.001
OCT signal index, mean (SD)	8.6 (0.5)	8.5 (0.5)	8.6 (0.5)	0.002
MoCA, score, mean (SD)	26.2 (3.0)	20.9 (2.4)	27.2 (1.8)	<0.001
MoCA, range	11-31	11-23	24-31	

*Data were presented as mean (SD) for continuous variables and number (percentage) for category variables. Cognitive impairment was defined as MoCA score < 24. FBG, fasting blood glucose; BMI, body mass index; TC, total cholesterol; HDL-C, high density liptein cholesterol; LDL-C, low density lipoprotein cholesterol; OCT, Optical coherence tomography; MoCA, Montreal Cognitive Assessment.*

Participants in the cognitive impairment group were older (*P* < 0.001) and less educated (*P* < 0.001), with lower incomes (*P* < 0.001), less myopia (*P* < 0.001), and more prevalent hypertension (*P* < 0.001), diabetes (*P* = 0.005), and dyslipidemia (*P* < 0.001). However, there were no significant differences between the two groups in terms of sex, current smoker and drinker status, their body mass index (BMI), and levels of TC, TG, HDL-C, and LDL-C.

### Ocular Characteristics of Participants

**FIGURE 2:**
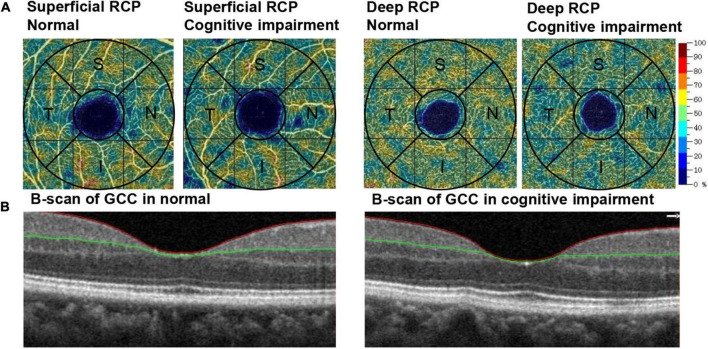
Example of OCTA-generated retinal measurements. **(A)** The color-coded vessel density maps of superficial RCP and deep RCP in participants with normal cognitive function and cognitive impairment. **(B)** B-scan of GCC in participants with normal cognitive function and cognitive impairment. RCP, retinal capillary plexus; GCC, ganglion cell complex; OCTA, optical coherence tomography angiography; S, superior; I, inferior; T, temporal; N, nasal.

**FIGURE 3:**
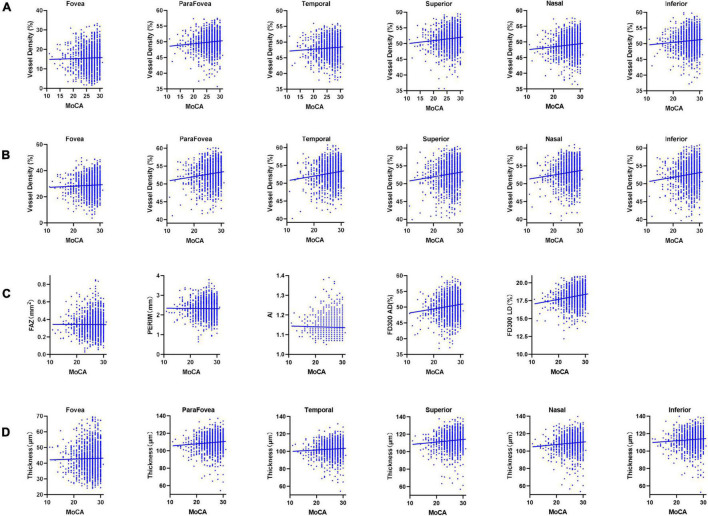
Scatter diagrams of the retinal measurements and cognitive function. **(A)** Superficial RCP vessel density. **(B)** Deep RCP vessel density. **(C)** FAZ, PERIM, AI, FD300 AD, and FD300 LD. **(D)** GCC thickness. RCP, retinal capillary plexus; FAZ, foveal avascular zone area; PERIM, foveal avascular zone perimeter; AI, acircularity index; FD300 AD, vessel area density within a 300 μm width ring surrounding the FAZ; FD300 LD, vessel length density within a 300 μm width ring surrounding the FAZ; GCC, ganglion cell complex; MoCA, Montreal Cognitive Assessment.

Figure 2 and Table 2 show the RCP and GCC characteristics measured using OCTA on the baseline survey. We observed significant differences between the two groups in terms of superficial RCP vessel density (parafovea: *P* = 0.003; temporal: *P* = 0.02; superior: *P* = 0.002; nasal: *P* = 0.007; inferior: *P* = 0.006), deep RCP vessel density (parafovea: *P* < 0.001; temporal: *P* < 0.001; superior: *P* < 0.001; nasal: *P* < 0.001; inferior: *P* < 0.001), FD300 AD (*P* < 0.001), and FD300 LD (*P* < 0.001). There were no significant differences between the groups for FAZ, PERIM, and AI. For GCC thickness, there were significant differences between groups (parafovea: *P* = 0.01; temporal: *P* = 0.04; superior: *P* = 0.003; nasal: *P* = 0.02; inferior: *P* = 0.01). Figure 3 shows the distributions of the RCP vessel density and GCC thickness according to the MoCA score.

**TABLE 2:** Ocular characteristics of eligible participants in the study.

Characteristics	Total (*n* = 1555)	Cognitive impairment (*n* = 268)	Normal (*n* = 1287)	*P*-value
**Superficial RCP, %, mean (SD)**				
Fovea	15.6 (5.7)	15.9 (5.8)	15.5 (5.7)	0.35
Parafovea	50.0 (2.9)	49.5 (2.8)	50.0 (2.9)	0.003
Temporal	48.1 (3.0)	47.7 (3.0)	48.2 (3.0)	0.02
Superior	51.6 (3.1)	51.0 (3.1)	51.7 (3.1)	0.002
Nasal	49.1 (3.1)	48.6 (3.0)	49.2 (3.2)	0.007
Inferior	50.9 (3.2)	50.5 (3.1)	51.0 (3.3)	0.006
**Deep RCP, %, mean (SD)**				
Fovea	28.7 (7.0)	28.9 (7.0)	28.7 (7.0)	0.79
Parafovea	52.8 (3.0)	52.2 (3.3)	53.0 (2.9)	<0.001
Temporal	52.8 (3.0)	52.2 (3.3)	53.0 (2.9)	<0.001
Superior	52.7 (3.5)	52.0 (3.7)	52.8 (3.4)	<0.001
Nasal	53.2 (3.1)	52.5 (3.4)	53.3 (3.0)	<0.001
Inferior	52.6 (3.5)	51.9 (3.7)	52.7 (3.4)	<0.001
FAZ, mm^2^, mean (SD)	0.3 (0.1)	0.3 (0.1)	0.3 (0.1)	0.14
PERIM, mm, mean (SD)	2.3 (0.4)	2.3 (0.4)	2.3 (0.4)	0.16
AI, mean (SD)	1.1 (0.0)	1.1 (0.0)	1.1 (0.0)	0.20
FD300 AD, %, mean (SD)	50.3 (3.5)	49.4 (3.8)	50.5 (3.4)	<0.001
FD300 LD, %, mean (SD)	18.1 (1.2)	17.7 (1.3)	18.2 (1.2)	<0.001
**GCC, μm, mean (SD)**				
Fovea	43.0 (8.4)	43.3 (8.3)	42.9 (8.4)	0.48
Parafovea	109.5 (8.9)	108.2 (9.5)	109.7 (8.8)	0.01
Temporal	102.6 (8.3)	101.7 (8.9)	102.8 (8.2)	0.04
Superior	112.9 (9.5)	111.3 (10.2)	113.2 (9.3)	0.003
Nasal	109.2 (9.7)	107.9 (10.3)	109.5 (9.6)	0.02
Inferior	113.2 (9.6)	111.9 (10.2)	113.5 (9.4)	0.01

*Data were presented as mean (SD) for continuous variables and number (percentage) for category variables. Cognitive impairment was defined as MoCA score < 24. RCP, retinal capillary plexus; FAZ, foveal avascular zone area; PERIM, foveal avascular zone perimeter; AI, acircularity index; FD300 AD, vessel area density within a 300 μm width ring surrounding the FAZ; FD300 LD, vessel length density within a 300 μm width ring surrounding the FAZ; GCC, ganglion cell complex.*

### Proportions of Cognitive Impairment in Participants Stratified by Sex, Diabetes, and Hypertension

Cognitive impairment was respectively observed in 16.7% and 17.9% of female and male participants, 24.4% and 16.2% of patients with and without diabetes, and 21.7% and 14.6% of patients with and without hypertension, and 14.9% and 43.8% of participants aged <65 and ≥65 (Supplementary Figure 1).

### Association of Retinal Capillary Plexus and Ganglion Cell Complex With Cognitive Impairment

We show the unadjusted and adjusted ORs of the RCP and GCC for cognitive impairment in Table 3. The adjusted OR with 95% CI for parafovea vessel density in deep RCP with cognitive impairment was 1.20 (1.03–1.39). Moreover, in regionals of temporal, superior, and inferior, the adjusted ORs with 95% CIs were 1.18 (1.01–1.37), 1.21 (1.05–1.40), and 1.17 (1.01–1.36), respectively. The adjusted ORs with 95% CIs for FD300 AD and FD300 LD with cognitive impairment were 1.23 (1.07–1.41) and 1.30 (1.13–1.49), respectively. The adjusted OR with 95%CI for thickness in the superior GCC with cognitive impairment was 1.16 (1.01–1.32).

**TABLE 3:** Association of retinal capillary plexus and ganglion cell complex with cognitive impairment in the logistic regression model.

Characteristics	OR (95%CI)	Adjusted OR (95%CI)	
**Superficial RCP, per 1 SD Decrease**			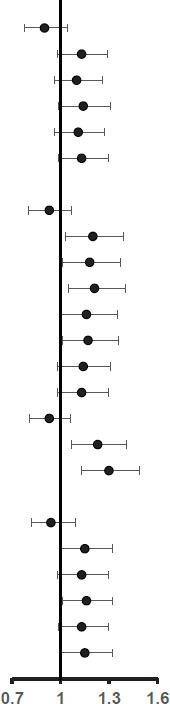
Fovea	0.94 (0.83–1.06)	0.90 (0.78–1.04)	
Parafovea	1.21 (1.06–1.37)	1.13 (0.98–1.29)	
Temporal	1.17 (1.03–1.33)	1.10 (0.96–1.26)	
Superior	1.22 (1.07–1.38)	1.14 (0.99–1.31)	
Nasal	1.19 (1.05–1.35)	1.11 (0.96–1.27)	
Inferior	1.19 (1.05–1.36)	1.13 (0.99–1.30)	
**Deep RCP, per 1 SD Decrease**			
Fovea	0.98 (0.86–1.12)	0.93 (0.80–1.07)	
Parafovea	1.29 (1.14–1.47)	1.20 (1.03–1.39)	
Temporal	1.29 (1.13–1.46)	1.18 (1.01–1.37)	
Superior	1.28 (1.12–1.45)	1.21 (1.05–1.40)	
Nasal	1.28 (1.13–1.46)	1.16 (1.00–1.35)	
Inferior	1.26 (1.10–1.43)	1.17 (1.01–1.36)	
FAZ, per 1 SD Decrease	1.11 (0.97–1.27)	1.14 (0.98–1.31)	
PERIM, per 1 SD Decrease	1.10 (0.96–1.25)	1.13 (0.98–1.30)	
AI, per 1 SD Increase	0.92 (0.81–1.05)	0.93 (0.81–1.06)	
FD300 AD, per 1 SD Decrease	1.33 (1.17–1.52)	1.23 (1.07–1.41)	
FD300 LD, per 1 SD Decrease	1.47 (1.29–1.68)	1.30 (1.13–1.49)	
**GCC, per 1 SD Decrease**			
Fovea	0.95 (0.84–1.09)	0.94 (0.82–1.09)	
Parafovea	1.18 (1.04–1.34)	1.15 (1.00–1.32)	
Temporal	1.15 (1.01–1.30)	1.13 (0.98–1.30)	
Superior	1.21 (1.07–1.37)	1.16 (1.01–1.32)	
Nasal	1.17 (1.03–1.33)	1.13 (0.99–1.30)	
Inferior	1.17 (1.03–1.33)	1.14 (1.00–1.31)	

*Adjusted for age, sex, educational status, income, refraction, hypertension, diabetes, and dyslipidemia. OR, odds ratio; CI, confidence interval; RCP, retinal capillary plexus; FAZ, foveal avascular zone area; PERIM, foveal avascular zone perimeter; AI, acircularity index; FD300 AD, vessel area density within a 300 μm width ring surrounding the FAZ; FD300 LD, vessel length density within a 300 μm width ring surrounding the FAZ; GCC, ganglion cell complex.*

In addition, we show the unadjusted and adjusted Betas of the RCP and GCC for cognitive function in Supplementary Table 1. The adjusted Betas with 95% CIs for each standard deviation decrease in vessel density of superficial and deep RCP (parafovea) with continuous MoCA score were –0.160 (–0.304 to –0.016) and –0.251 (–0.409 to –0.093), respectively. The adjusted Betas with 95% CIs for FD300 AD and FD300 LD with continuous MoCA score were –0.225 (–0.372 to –0.078) and –0.318 (–0.465 to –0.170), respectively. Further, the adjusted Beta with 95% CI for thickness in the parafovea GCC with continuous MoCA score was –0.205 (–0.351 to –0.059).

### Subgroup Analysis

We observed significant interactions of diabetes with GCC for cognitive impairment (Table 4). The ORs of the GCC items in participants with or without diabetes for cognitive impairment were 1.77 vs. 1.07 (P for interaction = 0.02) for parafovea, 1.71 vs. 1.05 (P for interaction = 0.02) for temporal, 1.74 vs. 1.07 (P for interaction = 0.01) for superior, 1.75 vs. 1.06 (P for interaction = 0.02) for nasal, and 1.70 vs. 1.08 (P for interaction = 0.04) for inferior. We did not find significant interactions of the GCC with sex, hypertension, or age for cognitive impairment. In addition, no interactions between RCP and sex, diabetes, hypertension, or age were observed for cognitive impairment (Tables 4, 5).

**TABLE 4:** Subgroup analyses of association of RCP and GCC with cognitive impairment based on sex, diabetes, and hypertension.

Characteristics	Sex			Diabetes			Hypertension		
	Male (*n* = 694)	Female (*n* = 861)		Diabetes (*n* = 193)	Non-Diabetes (*n* = 1362)		Hypertension (*n* = 575)	Non-Hypertension (*n* = 980)	
	Adjusted OR (95%CI)	Adjusted OR (95%CI)	*P*-value interaction	Adjusted OR (95%CI)	Adjusted OR (95%CI)	*P*-value interaction	Adjusted OR (95%CI)	Adjusted OR (95%CI)	*P*-value interaction
**Superficial RCP, per 1 SD Decrease**									
Fovea	0.89 (0.71–1.10)	0.91 (0.75–1.10)	0.94	1.18 (0.78–1.79)	0.87 (0.74–1.01)	0.15	0.93 (0.74–1.17)	0.88 (0.73–1.07)	0.77
Parafovea	1.26 (1.03–1.54)	1.01 (0.83–1.23)	0.13	1.22 (0.87–1.72)	1.11 (0.96–1.29)	0.60	1.22 (0.98–1.51)	1.07 (0.89–1.29)	0.35
Temporal	1.17 (0.96–1.42)	1.01 (0.83–1.24)	0.35	1.15 (0.83–1.61)	1.08 (0.93–1.26)	0.62	1.10 (0.90–1.36)	1.09 (0.91–1.31)	0.90
Superior	1.30 (1.07–1.58)	1.01 (0.82–1.23)	0.09	1.27 (0.91–1.77)	1.13 (0.97–1.31)	0.61	1.30 (1.05–1.61)	1.04 (0.87–1.26)	0.11
Nasal	1.26 (1.03–1.54)	0.98 (0.80–1.19)	0.09	1.19 (0.83–1.69)	1.09 (0.94–1.27)	0.66	1.22 (0.98–1.51)	1.03 (0.86–1.24)	0.24
Inferior	1.22 (1.01–1.48)	1.04 (0.85–1.27)	0.24	1.23 (0.87–1.73)	1.12 (0.96–1.30)	0.63	1.17 (0.94–1.45)	1.11 (0.93–1.33)	0.66
**Deep RCP, per 1 SD Decrease**									
Fovea	0.92 (0.73–1.16)	0.93 (0.77–1.13)	0.94	1.23 (0.81–1.85)	0.89 (0.76–1.04)	0.14	1.05 (0.83–1.33)	0.86 (0.72–1.04)	0.21
Parafovea	1.33 (1.08–1.63)	1.06 (0.84–1.33)	0.17	1.00 (0.69–1.45)	1.24 (1.05–1.47)	0.25	1.27 (1.02–1.59)	1.13 (0.92–1.39)	0.31
Temporal	1.27 (1.03–1.55)	1.07 (0.85–1.34)	0.31	1.09 (0.75–1.57)	1.19 (1.01–1.40)	0.61	1.20 (0.96–1.50)	1.15 (0.94–1.41)	0.56
Superior	1.34 (1.10–1.63)	1.07 (0.86–1.34)	0.15	0.99 (0.67–1.45)	1.25 (1.07–1.47)	0.21	1.33 (1.07–1.65)	1.11 (0.91–1.36)	0.17
Nasal	1.28 (1.05–1.57)	1.03 (0.82–1.29)	0.18	1.01 (0.70–1.46)	1.20 (1.02–1.42)	0.32	1.22 (0.98–1.51)	1.11 (0.90–1.37)	0.38
Inferior	1.28 (1.05–1.56)	1.06 (0.85–1.32)	0.23	0.91 (0.62–1.33)	1.23 (1.04–1.44)	0.15	1.23 (0.99–1.54)	1.12 (0.92–1.37)	0.35
FAZ, per 1 SD Decrease	1.15 (0.91–1.45)	1.12 (0.92–1.35)	0.93	0.89 (0.61–1.31)	1.18 (1.01–1.38)	0.19	0.98 (0.78–1.25)	1.24 (1.02–1.50)	0.16
PERIM, per 1 SD Decrease	1.13 (0.92–1.40)	1.12 (0.92–1.36)	0.94	0.88 (0.60–1.30)	1.17 (1.00–1.36)	0.19	0.96 (0.76–1.21)	1.25 (1.04–1.50)	0.08
AI, per 1 SD Decrease	0.94 (0.79–1.12)	0.92 (0.74–1.13)	0.90	0.97 (0.67–1.41)	0.92 (0.80–1.06)	0.74	0.95 (0.78–1.15)	0.92 (0.77–1.11)	0.92
FD300 AD, per 1 SD Decrease	1.32 (1.09–1.60)	1.12 (0.91–1.37)	0.32	1.18 (0.82–1.69)	1.23 (1.06–1.43)	0.94	1.12 (0.90–1.40)	1.29 (1.08–1.55)	0.47
FD300 LD, per 1 SD Decrease	1.35 (1.11–1.63)	1.24 (1.01–1.53)	0.62	1.43 (1.02–2.01)	1.28 (1.10–1.50)	0.52	1.29 (1.04–1.61)	1.30 (1.08–1.56)	0.81
**GCC, per 1 SD Decrease**									
Fovea	0.94 (0.77–1.15)	0.95 (0.77–1.17)	0.88	1.36 (0.91–2.02)	0.89 (0.76–1.04)	0.07	1.06 (0.84–1.33)	0.87 (0.72–1.06)	0.24
Parafovea	1.16 (0.96–1.41)	1.12 (0.91–1.36)	0.71	1.77 (1.21–2.59)	1.07 (0.92–1.25)	0.02	1.31 (1.06–1.62)	1.04 (0.86–1.26)	0.15
Temporal	1.17 (0.97–1.42)	1.05 (0.85–1.30)	0.41	1.71 (1.17–2.51)	1.05 (0.90–1.23)	0.02	1.29 (1.04–1.59)	1.02 (0.84–1.24)	0.15
Superior	1.17 (0.97–1.41)	1.13 (0.93–1.39)	0.83	1.74 (1.21–2.50)	1.07 (0.92–1.25)	0.01	1.31 (1.06–1.61)	1.06 (0.88–1.28)	0.18
Nasal	1.15 (0.95–1.40)	1.11 (0.91–1.35)	0.71	1.75 (1.20–2.55)	1.06 (0.91–1.24)	0.02	1.31 (1.06–1.62)	1.02 (0.85–1.24)	0.11
Inferior	1.13 (0.93–1.37)	1.13 (0.93–1.39)	0.91	1.70 (1.16–2.49)	1.08 (0.93–1.26)	0.04	1.27 (1.03–1.55)	1.06 (0.87–1.28)	0.23

*Adjusted for age, sex, educational status, income, refraction, hypertension, diabetes, and dyslipidemia. Subgroup associations were calculated from models that included interaction terms of factor x retinal measurements, and were adjusted for age, sex, educational status, income, refraction, hypertension, diabetes, and dyslipidemia. OR, odds ratio; CI, confidence interval; RCP, retinal capillary plexus; FAZ, foveal avascular zone area; PERIM, foveal avascular zone perimeter; AI, acircularity index; FD300 AD, vessel area density within a 300 μm width ring surrounding the FAZ; FD300 LD, vessel length density within a 300 μm width ring surrounding the FAZ; GCC, ganglion cell complex.*

**TABLE 5:** Subgroup analyses of association of RCP and GCC with cognitive impairment based on age.

Characteristics	Age		
	<65 (*n* = 1427)	≥65 (*n* = 128)	
	Adjusted OR (95%CI)	Adjusted OR (95%CI)	*P*-value interaction
**Superficial RCP, per 1 SD Decrease**			
Fovea	0.91 (0.78–1.06)	0.87 (0.59–1.28)	0.75
Parafovea	1.10 (0.95–1.28)	1.28 (0.82–1.99)	0.34
Temporal	1.06 (0.91–1.23)	1.26 (0.84–1.89)	0.27
Superior	1.14 (0.99–1.32)	1.12 (0.72–1.74)	0.94
Nasal	1.07 (0.92–1.24)	1.34 (0.86–2.10)	0.20
Inferior	1.11 (0.96–1.29)	1.19 (0.79–1.79)	0.48
**Deep RCP, per 1 SD Decrease**			
Fovea	0.91 (0.77–1.06)	1.07 (0.71–1.61)	0.39
Parafovea	1.21 (1.03–1.43)	1.08 (0.72–1.62)	0.99
Temporal	1.19 (1.01–1.40)	1.01 (0.68–1.49)	0.90
Superior	1.23 (1.05–1.44)	1.09 (0.74–1.60)	0.86
Nasal	1.16 (0.98–1.37)	1.14 (0.79–1.66)	0.78
Inferior	1.19 (1.01–1.40)	1.04 (0.70–1.55)	0.94
FAZ, per 1 SD Decrease	1.15 (0.98–1.35)	1.02 (0.70–1.50)	0.61
PERIM, per 1 SD Decrease	1.15 (0.99–1.34)	0.99 (0.68–1.45)	0.47
AI, per 1 SD Decrease	0.94 (0.81–1.10)	0.90 (0.65–1.24)	0.54
FD300 AD, per 1 SD Decrease	1.20 (1.04–1.40)	1.26 (0.86–1.83)	0.59
FD300 LD, per 1 SD Decrease	1.28 (1.10–1.48)	1.37 (0.89–2.09)	0.52
**GCC, per 1 SD Decrease**			
Fovea	0.93 (0.79–1.09)	1.08 (0.72–1.61)	0.61
Parafovea	1.10 (0.95–1.28)	1.51 (0.97–2.36)	0.18
Temporal	1.08 (0.93–1.26)	1.36 (0.91–2.03)	0.27
Superior	1.11 (0.96–1.29)	1.59 (0.97–2.60)	0.17
Nasal	1.09 (0.94–1.27)	1.52 (0.93–2.50)	0.16
Inferior	1.11 (0.95–1.29)	1.33 (0.91–1.92)	0.38

*Adjusted for age, sex, educational status, income, refraction, hypertension, diabetes, and dyslipidemia. Subgroup associations were calculated from models that included interaction terms of factor x retinal measurements, and were adjusted for age, sex, educational status, income, refraction, hypertension, diabetes, and dyslipidemia. OR, odds ratio; CI, confidence interval; RCP, retinal capillary plexus; FAZ, foveal avascular zone area; PERIM, foveal avascular zone perimeter; AI, acircularity index; FD300 AD, vessel area density within a 300 μm width ring surrounding the FAZ; FD300 LD, vessel length density within a 300 μm width ring surrounding the FAZ; GCC, ganglion cell complex.*

## Discussion

To our knowledge, this was the first study to identify an association between the RCP and cognitive impairment in a large community-based population using OCTA. We found that lower RCP vessel densities, lower FD300 AD and LD, and thinner GCC were associated with cognitive impairment. Furthermore, the magnitude of the association between lower RCP and thinner GCC with cognitive impairment showed consistent connections to subgroups based on sex, hypertension, and age, although diabetes only impacted the association of the GCC with cognitive impairment.

Collectively, studies to date have not shown a clear association between the RCP and cognitive impairment using OCTA (Cheung et al., 2021; Rifai et al., 2021). Our findings showed that the vessel densities in the deep RCP, but not in the superficial RCP, were significantly associated with cognitive impairment. These findings were consistent with those of several previous studies that found a reduction in the RCP in AD groups (Bulut et al., 2018; Jiang et al., 2018b); however, several studies have reported conflicting results (Haan et al., 2019; Querques et al., 2019; Yoon et al., 2019; van de Kreeke et al., 2020). Furthermore, it is interesting to note that FD300 AD and LD were associated with cognitive impairment in our study. FD300 AD and LD represent the vascular conditions surrounding the FAZ. Their associations with cognitive impairment were firstly revealed in our study and should be further verified in future studies. However, in our study, we found that FAZ, PERIM, and AI were not associated with cognitive impairment. This is in line with previous results showing that the FAZ is not significantly associated with cognitive impairment (Haan et al., 2019; Chua et al., 2020; van de Kreeke et al., 2020), and these results contradict other findings (Bulut et al., 2018; O’Bryhim et al., 2018). This pattern of inconsistent findings may be explained by previous studies not accounting for the different disease stages or having included relatively small samples with limited adjustment for potential confounders (Zhang et al., 2020). However, it is important to point out that the difference of the RCP vessel density thickness between cognitive impairment and normal was mild, although it was statistically significant. Potentially, the reduced RCP in participants with cognitive impairment may be explained by pathogenic Aβ deposits and pericyte loss in retinal blood vessels (Shi et al., 2020). Furthermore, the association found in the deep RCP, rather than in the superficial RCP, may be explained by the superficial RCP blood supply comprising larger diameter vessels compared with deep RCP vessels; the deep RCP might be affected earlier and to a greater extent than the superficial RCP (Wang et al., 2018).

In our study, the thinning thickness of the superior GCC was associated with cognitive impairment. In support of our findings, Hinton et al. (1986) previously showed an association between a thinner RNFL and dementia, and several large community-based cohort studies have also reported an association between a thinner RNFL or GCC and cognitive impairment (Ko et al., 2018; Ito et al., 2020; Ward et al., 2020). In our study, we also showed that the thickness of the GCC was associated with cognitive impairment, further adding to the literature. As we know, the connection between the integrity of the retina and functionality of its vasculature is linked intimately, as greater vascular risks could increase the risk for ocular diseases, such as glaucoma (Choi and Kook, 2015). However, the exact mechanisms by which the retinal vascular exerts its benefits in the retinal structure are not established. Therefore, further studies should assess the association between the RCP and GCC to interpret the effect of impaired retinal microvasculature on retinal neurodegeneration.

The stronger association of cognitive impairment was observed in our study with GCC in the subgroup of participants with diabetes. The associations of the RCP and GCC with cognitive impairment were consistent across the subgroups based on sex, hypertension, and age. Therefore, the study raised the possibility that diabetes enhances the association of the GCC with cognitive impairment but not RCP. This should be further assessed in future studies.

The main strengths of this study were the use of detailed ophthalmic examinations and testing cognitive function in a community-based sample. In this study, the JECS is advantageous in that it included a relatively large sample size; thus, our study has the strength of adjusting for several potential confounding factors (e.g., hypertension, diabetes, and refraction) and performing subgroup analyses. Nevertheless, there are some limitations to our study. First, this cross-sectional study cannot indicate a causal inference. It would be of interest to explore the alterations of cognitive function in relation to vascular and ganglion cells over time, and this may be a future study objective as a follow-up to the current cohort. Second, the study sample was from the Jidong communities, and this cannot adequately represent other populations. Third, no markers of neurodegenerative disease was a limitation of our study. Due to their invasive, time consuming, and high cost, biomarker tests like amyloid PET neuroimaging or CSF testing are limited in their availability in a community. Although, there is no doubt that markers of neurodegenerative disease are of great importance to support our findings. Finally, we recognize that statistical significance is not equivalent to clinical relevance. Thus, the study findings should be interpreted carefully for application in clinical practice, and further studies using artificial intelligence or combining other biomarkers are necessary.

## Conclusion

We found a significant association between lower RCP vessel density and thinner GCC thickness with cognitive impairment. These associations were not altered by sex, hypertension, or age, but strengthened in participants with diabetes. Our results support the potential use of OCT/OCTA measurement, which is a low-cost, widely available, non-invasive, rapid method for the detection of cognitive impairment.

## Data Availability Statement

The raw data supporting the conclusions of this article will be made available by the authors, without undue reservation.

## Ethics Statement

The studies involving human participants were reviewed and approved by the Ethics Committee of Staff Hospital of Jidong oil-field of Chinese National Petroleum (approval document 2018 YILUNZI 1). The patients/participants provided their written informed consent to participate in this study.

## Author Contributions

ML, JQ, and FL designed and conceptualized the study and interpreted the data. KY, XC, and JZ analyzed the data. KY, CY, XZ, YX, BS, CL, and KS had a major role in the acquisition of data. KY, LC, and XC drafted the manuscript. CY, ML, JQ, and FL revised the manuscript for intellectual content. All authors read and approved the final manuscript.

## Conflict of Interest

The authors declare that the research was conducted in the absence of any commercial or financial relationships that could be construed as a potential conflict of interest.

## Publisher’s Note

All claims expressed in this article are solely those of the authors and do not necessarily represent those of their affiliated organizations, or those of the publisher, the editors and the reviewers. Any product that may be evaluated in this article, or claim that may be made by its manufacturer, is not guaranteed or endorsed by the publisher.

## Supplementary Material

Supplementary Figure 1 Proportions of cognitive impairment in participants stratified by sex, diabetes, hypertension, and age.

## Abbreviations

AD: Alzheimer’s disease
AI: acircularity index
BMI: body mass index
Cis: confidence intervals
FAZ: foveal avascular zone area
FBG: fasting blood glucose
FD300 AD: vessel area density within a 300 μm width ring surrounding the FAZ
FD300 LD: vessel length density within a 300 μm width ring surrounding the FAZ
GCC: ganglion cell complex
HDL-C: high density lipoprotein
LDL-C: low-density lipoprotein
MoCA: the Montreal Cognitive Assessment
OCT: optical coherence tomography
OCTA: optical coherence tomography angiography
ORs: odds ratios
PERIM: foveal avascular zone perimeter
RCP: retinal capillary plexus
RNFL: retinal nerve fiber layer
TC: total cholesterol
TG: triglyceride.
