# Supplementary material for: Decreased Vessel Density in Retinal Capillary Plexus and Thinner Ganglion Cell Complex Associated With Cognitive Impairment
Source: Front Aging Neurosci. 2022 Apr 26;14:872466. doi: 10.3389/fnagi.2022.872466 (PMC9087336; doi:10.3389/fnagi.2022.872466)
Supplement: Supplementary file 2 [file Table_1.DOCX]

**Supplementary Table 1. Association of retinal capillary plexus and ganglion cell complex with cognitive function in the linear regression model**

| **Characteristics** | **Beta (95%CI)** | **Adjusted Beta (95%CI)** |  |
| --- | --- | --- | --- |
| Superficial RCP, per 1 SD Decrease |  |  |  |
| Fovea | -0.079 (-0.231 – 0.073) | -0.053 (-0.200 – 0.095) |  |
| Parafovea | -0.279 (-0.430 – -0.128) | -0.160 (-0.304 – -0.016) |  |
| Temporal | -0.202 (-0.353 – -0.050) | -0.092 (-0.236 – 0.052) |  |
| Superior | -0.297 (-0.448 – -0.146) | -0.182 (-0.327 – -0.038) |  |
| Nasal | -0.288 (-0.439 – -0.137) | -0.170 (-0.314 – -0.025) |  |
| Inferior | -0.241 (-0.393 – -0.090) | -0.145 (-0.288 – -0.001) |  |
| Deep RCP, per 1 SD Decrease |  |  |  |
| Fovea | -0.136 (-0.288 – 0.016) | -0.092 (-0.240 – 0.056) |  |
| Parafovea | -0.389 (-0.540 – -0.238) | -0.251 (-0.409 – -0.093) |  |
| Temporal | -0.410 (-0.560 – -0.259) | -0.264 (-0.421 – -0.107) |  |
| Superior | -0.341 (-0.492 – -0.190) | -0.224 (-0.377 – -0.070) |  |
| Nasal | -0.374 (-0.526 – -0.223) | -0.205 (-0.364 – -0.046) |  |
| Inferior | -0.348 (-0.499 – -0.197) | -0.226 (-0.381 – -0.072) |  |
| FAZ, per 1 SD Decrease | 0.009 (-0.143 – 0.161) | 0.016 (-0.132 – 0.164) |  |
| PERIM, per 1 SD Decrease | 0.028 (-0.124 – 0.181) | 0.031 (-0.115 – 0.178) |  |
| AI, per 1 SD Decrease | 0.085 (-0.067 – 0.237) | 0.067 (-0.076 – 0.210) |  |
| FD300 AD, per 1 SD Decrease | -0.382 (-0.533 – -0.232) | -0.225 (-0.372 – -0.078) |  |
| FD300 LD, per 1 SD Decrease | -0.533 (-0.683 – -0.384) | -0.318 (-0.465 – -0.170) |  |
| GCC, per 1 SD Decrease |  |  |  |
| Fovea | -0.063 (-0.316 – 0.089) | -0.083 (-0.233 – 0.067) |  |
| Parafovea | -0.262 (-0.414 – -0.110) | -0.205 (-0.351 – -0.059) |  |
| Temporal | -0.195 (-0.347 – -0.043) | -0.160 (-0.306 – -0.013) |  |
| Superior | -0.293 (-0.444 – -0.141) | -0.210 (-0.355 – -0.065) |  |
| Nasal | -0.278 (-0.430 – -0.126) | -0.223 (-0.369 – -0.077) |  |
| Inferior | -0.220 (-0.372 – -0.068) | -0.175 (-0.321 – -0.030) |  |
|  |  |  |  |
|  |  |  |  |

Adjusted for age, sex, educational status, income, refraction, hypertension, diabetes, and dyslipidemia.

CI, confidence interval; RCP, retinal capillary plexus; FAZ, foveal avascular zone area; PERIM, foveal avascular zone perimeter; AI, acircularity index; FD300 AD, vessel area density within a 300 µm width ring surrounding the FAZ; FD300 LD, vessel length density within a 300 µm width ring surrounding the FAZ; GCC, ganglion cell complex.
